# Advances in research on the relationship between mitochondrial function and colorectal cancer: a bibliometric study from 2013 to 2023

**DOI:** 10.3389/fimmu.2024.1480596

**Published:** 2024-11-13

**Authors:** Jinhui Liu, Yonglong Chang, Qinling Ou, Linzi Chen, Haixia Yan, Duanyang Guo, Chongjie Wang, Sifang Zhang

**Affiliations:** ^1^ College of Integrated Traditional Chinese & Western Medicine, Hunan University of Traditional Chinese Medicine, Changsha, Hunan, China; ^2^ Department of Integrated Traditional Chinese & Western Medicine, The Second Xiangya Hospital, Central South University, Changsha, Hunan, China; ^3^ Department of Addiction Medicine, Hunan Institute of Mental Health, Brain Hospital of Hunan Province (The Second People’s Hospital of Hunan Province), Changsha, Hunan, China

**Keywords:** mitochondria, colorectal cancer, apoptosis, oxidative stress, mitochondrial metabolism, tumor microenvironment, tumor immunology

## Abstract

The study provides a thorough examination of literature from 2013 to 2023, delving into the intricate relationship between mitochondrial function and colorectal cancer (CRC). It offers a concise overview of the current landscape and emerging trends in this rapidly evolving research area. The findings indicate a consistent rise in annual publications, reflecting growing interest and significant potential in the field. China emerges as the leading contributor, followed by the United States and India. However, despite China’s dominance in output, its average citation rate is lower than that of the US, which leads in citations per publication, highlighting a noticeable disparity. In the realm of research institutions, Shanghai Jiao Tong University and China Medical University are identified as major contributors, yet the potential for inter-institutional collaboration remains largely untapped, suggesting avenues for future synergy. Internationally, China-US collaborations are particularly robust, fostering cross-border knowledge exchange. Hyun Jin Won and Li Wei are recognized as prolific authors, while Ahmedin Jemal is an influential co-cited scholar, noted for his seminal contributions. Keyword analysis reveals research focus areas, such as the complex CRC tumor microenvironment, molecular mechanisms of oxidative stress, and key multidrug resistance pathways. It also highlights the promising potential of mitochondria-targeted therapies and nanomolecular technologies in clinical practice, signaling their growing significance in addressing complex health challenges. The study underscores the imperative to validate complex mitochondrial mechanisms and signaling pathways in CRC, with a particular emphasis on translating these insights into drug targets for clinical trials. Advancing this research is expected to refine and enhance CRC treatment strategies. Additionally, it highlights the urgency of validating mitochondrial complexities in CRC, advocating for collaborative efforts to link these mechanisms with tailored therapeutic interventions for clinical testing. This integrated approach promises significant advancements in developing effective, targeted CRC treatments, ultimately improving patient outcomes.

## 1 Introduction

Colorectal cancer (CRC), a prevalent malignancy of the digestive tract, originates from the epithelial cells of the large intestine mucosa. Its multifaceted etiology involves a range of factors, including age, gender, genetic predispositions, environmental influences, and lifestyle choices. Epidemiological studies indicate that CRC ranks third in incidence and second in mortality among all cancers worldwide, with a concerning trend toward younger demographics. Projections suggest that by 2030, one in ten patients with colon cancer will be under 50 years old, and one in four patients with rectal cancer will fall within the same age group (1, 2).

Despite the widespread use of current treatments such as surgery, chemotherapy, and radiotherapy, their effectiveness remains suboptimal, often hampered by multidrug resistance and significant toxic side effects. Against this backdrop, mitochondria, the cellular energy converters, have garnered substantial attention due to their pivotal roles in cell metabolism, signal transduction, apoptosis regulation, and cell cycle control (3). Notably, the essential function of mitochondria in the metabolic reprogramming, proliferation, survival, and metastasis of tumor cells offers a novel therapeutic perspective (4).

The link between mitochondrial dysfunction and CRC progression is well-established, making the investigation of this relationship essential for CRC prevention and treatment. Numerous studies have demonstrated that imbalances in mitochondrial-related tumor markers can predict tumor progression and prognosis (5). Mitochondria-targeted therapies, including tumor-specific treatments, immune modulation, and the reversal of multidrug resistance, hold significant promise.

In recent years, the research focus on CRC and mitochondrial function has intensified, reflected in the growing volume of related literature. However, from a bibliometric standpoint, the research in this domain remains insufficiently explored. Bibliometrics, a methodology introduced by Pritchard in 1969, examines the internal connections within academic publications through mathematical and statistical approaches (6). It identifies publication trends by country, institution, author, and journal, assesses their quality and impact, and uncovers interrelationships within the data.

This study employs tools such as CiteSpace and VOSviewer for an in-depth bibliometric analysis of the literature published between 2013 and 2023 on the relationship between CRC and mitochondria. Through visual analysis, key data are extracted and represented in intuitive graphs and charts, facilitating the identification of research hotspots and emerging trends. Additionally, the study reveals co-citation relationships within the literature, offering researchers deeper insights into the knowledge structure and thematic evolution of this field (7, 8).

By employing bibliometric methods to analyze the literature from 2013 to 2023 concerning CRC and mitochondrial function, this study provides a comprehensive overview of the current developments and predicts future research directions and focal points. These findings offer critical references and guidance for subsequent research, with the expectation that future studies will further elucidate the intrinsic link between CRC and mitochondrial function, contributing innovative strategies and approaches for CRC treatment and prevention.

## 2 Methods

### 2.1 Data collection

**Figure 1:**
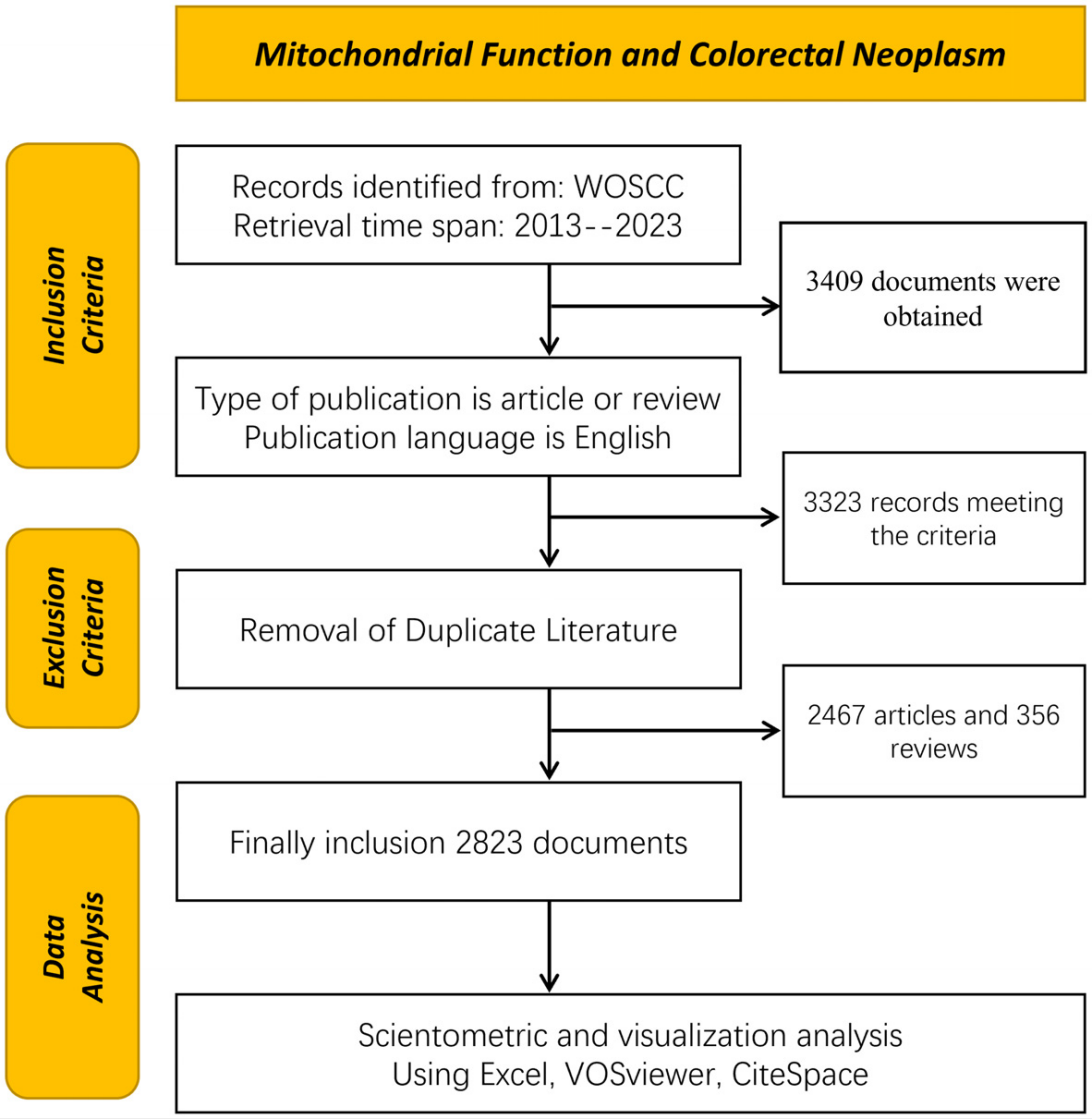
Flow chart for search strategy of publications.

The bibliometric analysis, leveraging the Web of Science Core Collection (WoSCC), provides high-quality literature data on the relationship between colorectal tumors and mitochondria. This analysis encompasses literature published from January 1, 2013, to December 31, 2023, aiming to comprehensively reflect the latest advancements in this field. All data retrieval was finalized on April 20, 2024, ensuring both accuracy and completeness. The search terms used were TS=(“Rectal Neoplasm” OR “Rectal Tumor” OR “Rectal Cancer” OR “Rectum Neoplasm” OR “Rectum Cancer” OR “Cancer of the Rectum” OR “Cancer of Rectum” OR “Colorectal Neoplasm” OR “Colorectal Tumor” OR “Colorectal Cancer” OR “Colorectal Carcinoma” OR “Colonic Neoplasm” OR “Colon Neoplasm” OR “Cancer of Colon” OR “Colon Cancer” OR “Cancer of the Colon” OR “Colonic Cancer”) TS=(“Mitochondria” OR “Mitochondrion” OR “Mitochondrial” OR “Mitophagy”). A total of 3,409 articles were retrieved, which were subsequently screened by two researchers based on the following criteria: 1) exclusion of non-English articles; 2) exclusion of literature not classified as “Article” and “Review”; 3) exclusion of non-SCI-E indexed literature. After this screening process, 2,823 documents were included and exported in document format for further analysis. The screening criteria are detailed in **Figure 1**.

### 2.2 Data analysis tools

This study employed two leading bibliometric tools, CiteSpace and VOSviewer, to process and visualize the data. CiteSpace is particularly adept at uncovering co-citation networks and keyword co-occurrence networks and identifying hot topics and trends within research fields. In contrast, VOSviewer excels in constructing collaboration networks among countries/regions, institutions, journals, and authors, and in presenting the co-occurrence relationships of keywords.

### 2.3 Data analysis

The collected literature underwent rigorous quantitative and qualitative analyses. The quantitative analysis focused on the top 10 authors, journals, institutions, and countries/regions with the highest publication counts, aiming to reveal their influence within the field. The qualitative analysis explored the relationships between authors and their co-cited counterparts, the interactions between journals and their co-cited academic journals, the content of co-cited references, and the co-occurrence patterns of keywords.

### 2.4 Visual analysis

To visually depict research trends, collaboration networks, and keyword co-occurrence networks, we extensively utilized the visual charts generated by CiteSpace and VOSviewer. These visualizations not only enhance the clarity of the data analysis but also enable readers to quickly grasp complex data relationships.

### 2.5 Analysis of research trends

Analysis of keyword timelines and the citation strength of co-cited literature enabled the identification of current research hotspots and facilitated well-grounded predictions of future research trends. These forecasts offer substantial reference value, guiding the direction and focus of subsequent research efforts.

## 3 Results

### 3.1 Annual trends in publications

This study examined 2,823 papers on the relationship between mitochondrial function and CRC, contributed by 17,243 researchers from 3,204 organizations across 94 countries. These papers, published in 757 specialized journals, extensively cited 128,167 related articles from 8,947 journals.

Through stringent search criteria and within a defined time span, publications related to mitochondrial function and CRC research were retrieved from the Web of Science Core Collection (WoSCC) database. Research articles dominate the dataset, comprising 2,467 papers (87.39%), while review articles account for 356 (12.61%), offering a wealth of research findings and academic insights in this field.

**Figure 2:**
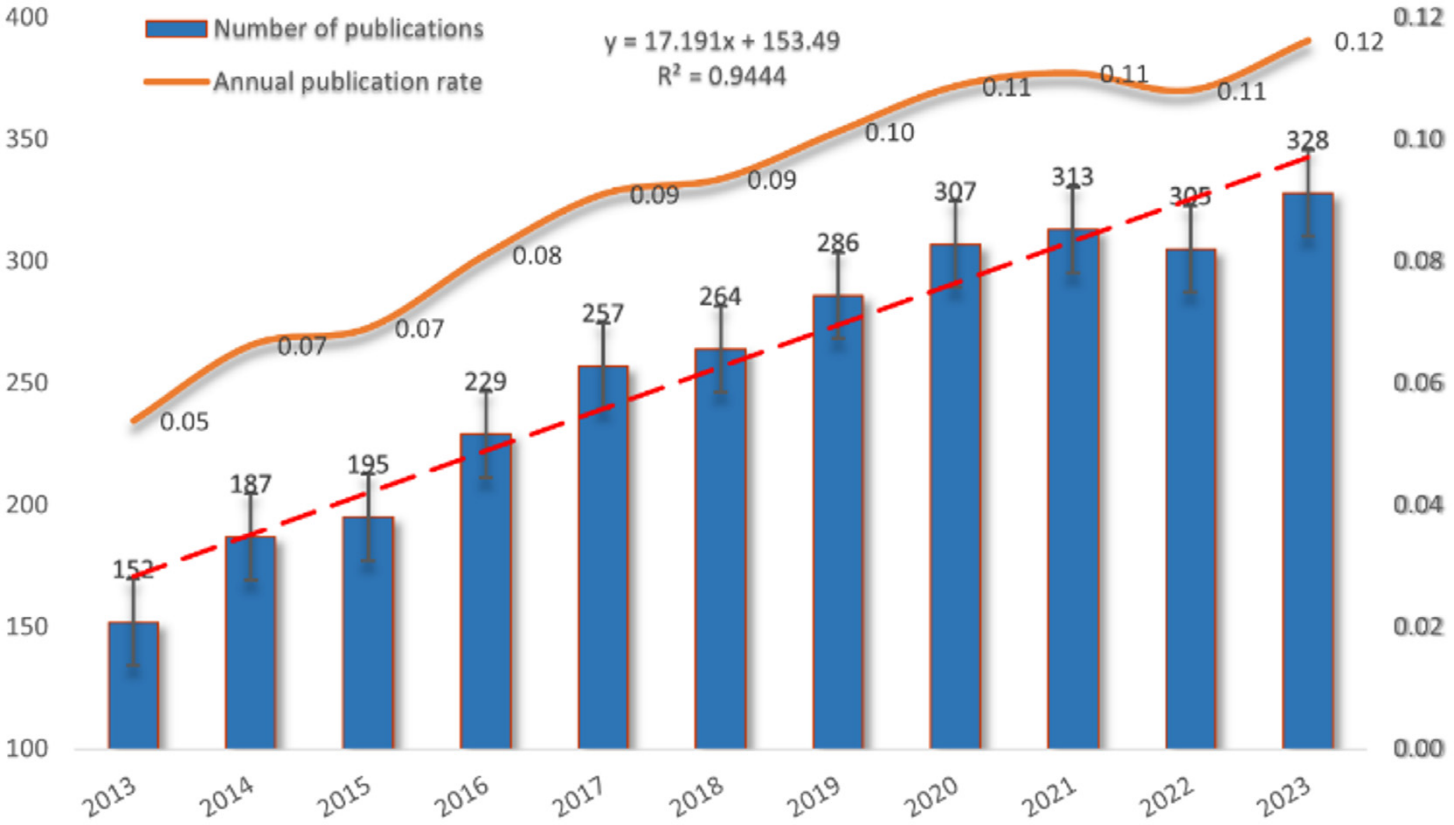
The annual growth trends of publication during the period 2013-2023.

**Figure 2** illustrates the publication trends in this field over the past decade, revealing a steady and consistent rise in annual publications related to mitochondrial function and CRC research since 2013. This trend highlights the increasing activity and growing interest in the area. After data fitting, a strong correlation between publication year and annual publication volume (R2 = 0.9444) is confirmed, further validating the ongoing development and vitality of research in this domain.

### 3.2 Country

**Figure 3:**
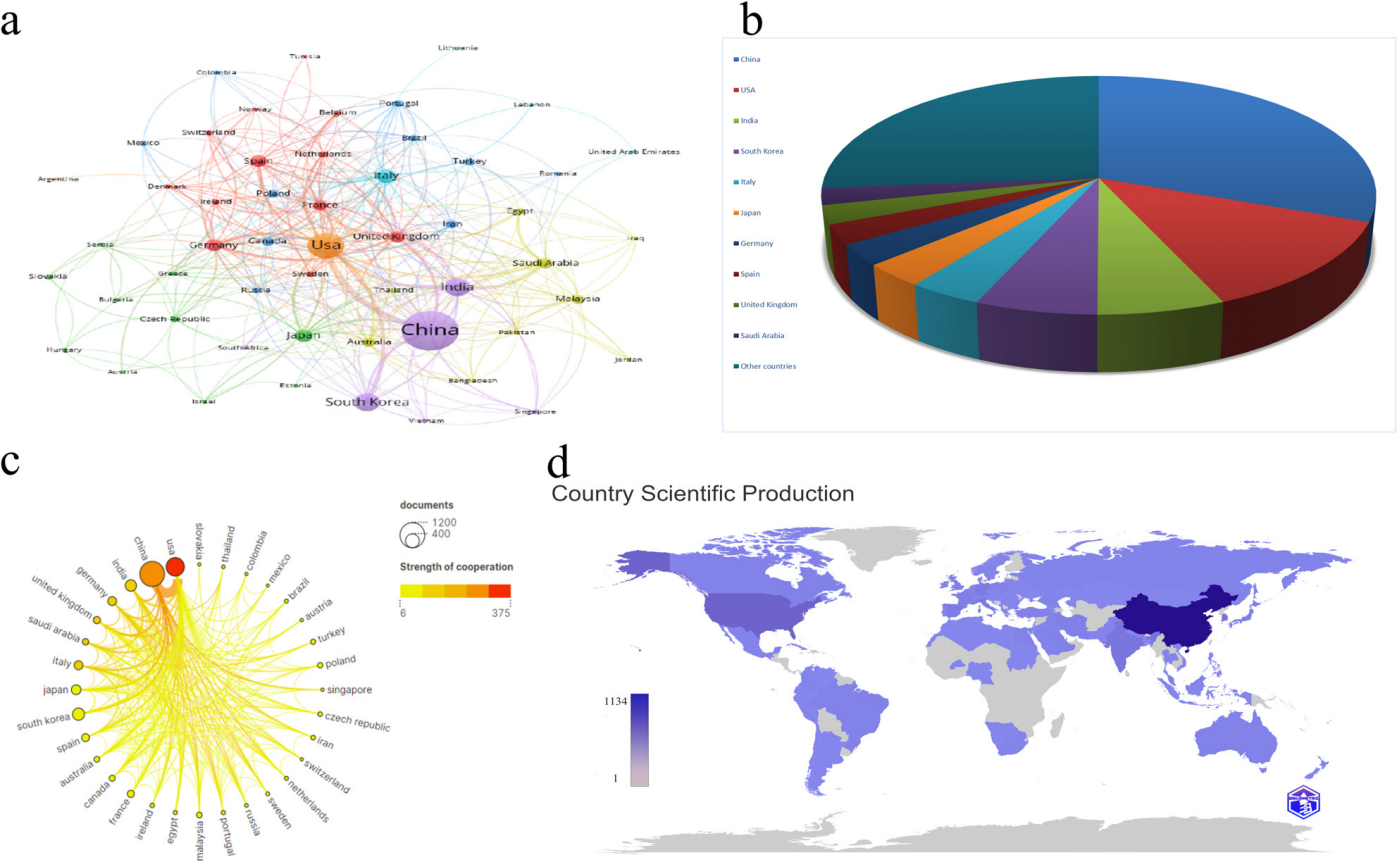
**(A)** Cooperation network of countries/regions in the field. **(B)** Contribution Rate of Publications from the Top 10 Countries/Regions. **(C)** Cooperation network among top 20 countries/regions based on quantity of publications. **(D)** Country/region Scientific Production and Collaboration Map. The color deepens as the number of literature published by a country increases.

Between 2013 and 2023, 54 countries engaged in extensive research on the relationship between mitochondrial function and CRC. As detailed in **Table 1**, China’s contribution is particularly noteworthy, leading with 1,134 publications (30.21%), followed by the United States with 468 publications (12.47%), India with 239 publications (6.37%), South Korea with 229 publications (6.10%), and Italy with 139 publications (3.70%). China also leads in total citations, with 27,312, significantly surpassing other countries. The United States follows with 19,982 citations, and India with 5,674 citations. Notably, the United States and the United Kingdom rank highest in average citation rates, at 42.70 and 39.49 respectively, reflecting the high quality and substantial impact of research in these two nations. To illustrate the international collaboration network in mitochondrial function and CRC research, the Bibliometrics online analysis platform and CiteSpace were employed for network visualization. **Figure 3A** differentiates countries by color and area size, representing publication numbers, with lines indicating collaboration. **Figure 3B** depict the proportion of publication quantities among different countries. In **Figure 3C**, color intensity represents the volume of publications, while connecting lines indicate collaborative efforts, with thicker lines signifying more frequent partnerships. **Figure 3D** depict the number of publications among different countries. The figures reveal that China and the United States are the most prolific contributors, while the United States, Germany, and the United Kingdom show highly active collaborations due to their central roles. These analyses offer valuable insights into international cooperation in mitochondrial function and CRC research and provide robust data to support future collaborative efforts.

**Table 1:** Top 10 active countries/regions.

Rank	country	documents	citations	Average citation/publication
1	China	1134	27312	24.1
2	USA	468	19982	42.7
3	India	239	5674	23.7
4	South Korea	229	5239	22.9
5	Italy	139	3929	28.3
6	Japan	117	3138	26.8
7	Germany	106	2992	28.2
8	Spain	100	2784	27.8
9	United Kingdom	93	3673	39.5
10	Saudi Arabia	84	1373	16.3

### 3.3 Organization

**Figure 4:**
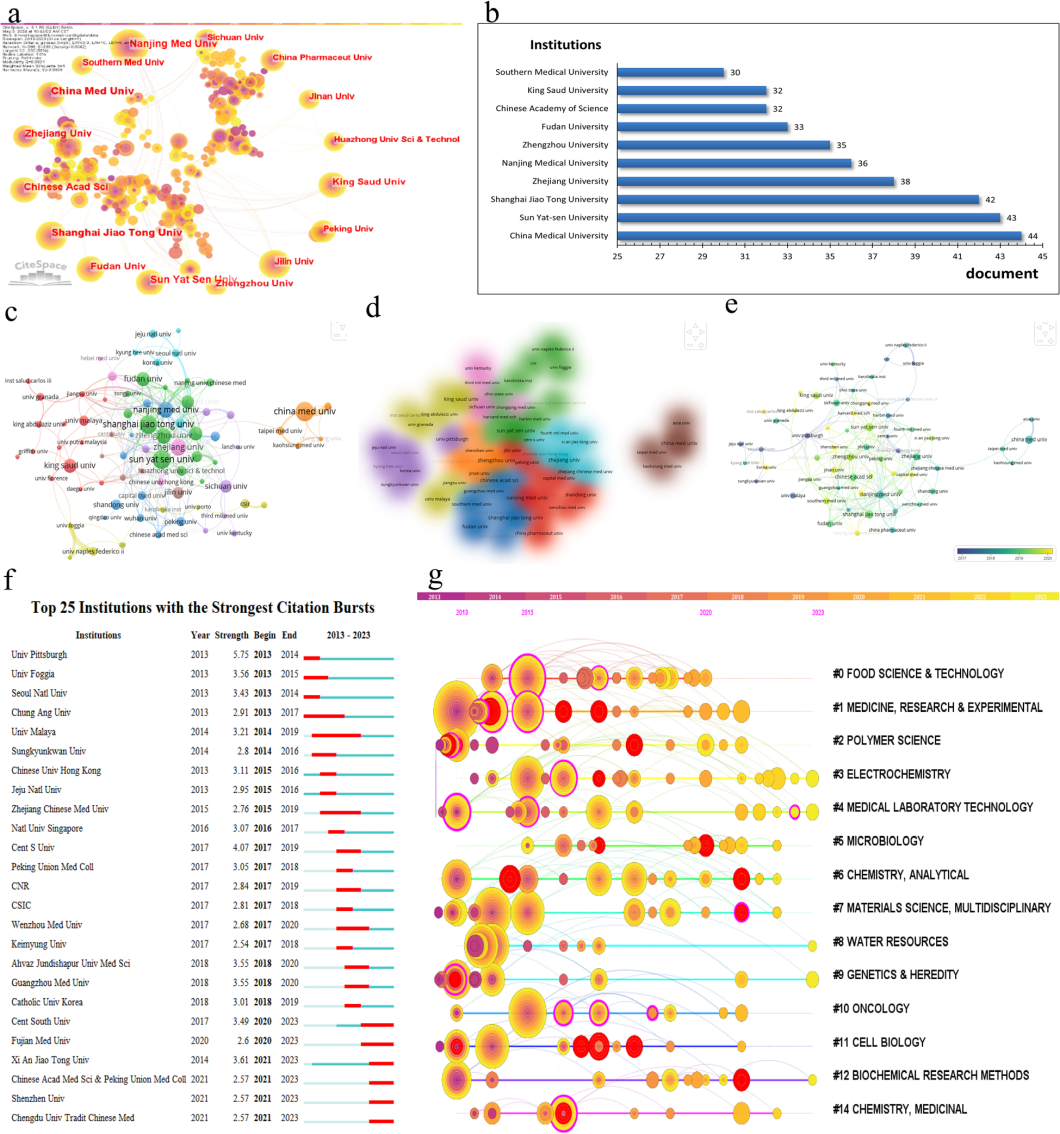
**(A)** Cooperation network of institutions and institutional label clustering based on LLR algorithm in the field. **(B)** Top 10 active institutions in the field. **(C)** Cooperation network visualization of institutions in the field. **(D)** Cooperation overlay visualization of institutions in the field. **(E)** Cooperation density visualization of institutions in the field. **(F)** Top 25 institutions with the strongest citation bursts. **(G)** Timeline clustering map of institutional cooperation networks by CiteSpace.

In the research domain of CRC and mitochondria, 3,204 institutions worldwide have actively participated. **Figure 4A** depicts the research productivity levels of institutions engaged in this field of study, as well as their inter-institutional collaborative partnerships. **Figure 4B** and **Table 2** spotlight the top ten institutions contributing most significantly to this area. China Med University lead the list with 44 publications, followed by Sun Yat-sen University and Shanghai Jiao Tong University, with 43 publications and 42 publications. Notably, eight of these top ten institutions are based in China, underscoring the country’s robust research capabilities in this field. To better understand the collaboration networks among these institutions, VOSviewer and CiteSpace were employed for network visualization analysis. As illustrated in **Figures 4C–E**, 61 institutions that have published at least three papers and have established collaborative relationships with other institutions were clustered into nine groups. Despite 95 institutions meeting the collaboration threshold, the overall level of collaboration remains relatively low, indicating a fragmented nature in research partnerships within this field in **Figure 4F**. **Figure 4G** further visualize institutional co-occurrence and clustering. The cluster analysis identifies seven primary research groups: #0 Food Science and Technology; #1 Medicine, Research and Experiment; #2 Polymer Science; #3 Electrochemistry; #4 Medical Laboratory Technology; #9 Genetics and Heredity; and #11 Cell Biology in **Figure 4G**. These clusters highlight the diversity of research within this field and demonstrate the specialized expertise of different institutions across various research directions. These analyses offer a more comprehensive understanding of the current status and emerging trends in CRC and mitochondria research.

**Table 2:** Top 10 active institutions.

Rank	organization	documents	citations	Average citation/publication
1	china med univ	44	1513	34.4
2	sun yat sen univ	43	1219	28.3
3	shanghai jiao tong univ	42	1348	32.1
4	zhejiang univ	38	1132	29.8
5	nanjing med univ	36	1846	51.3
6	zhengzhou univ	35	753	21.5
7	fudan univ	33	777	23.5
8	chinese acad sci	32	628	19.6
9	king saud univ	32	635	19.8
10	southern med univ	30	590	19.7

### 3.4 Authors and co-cited authors

**Figure 5:**
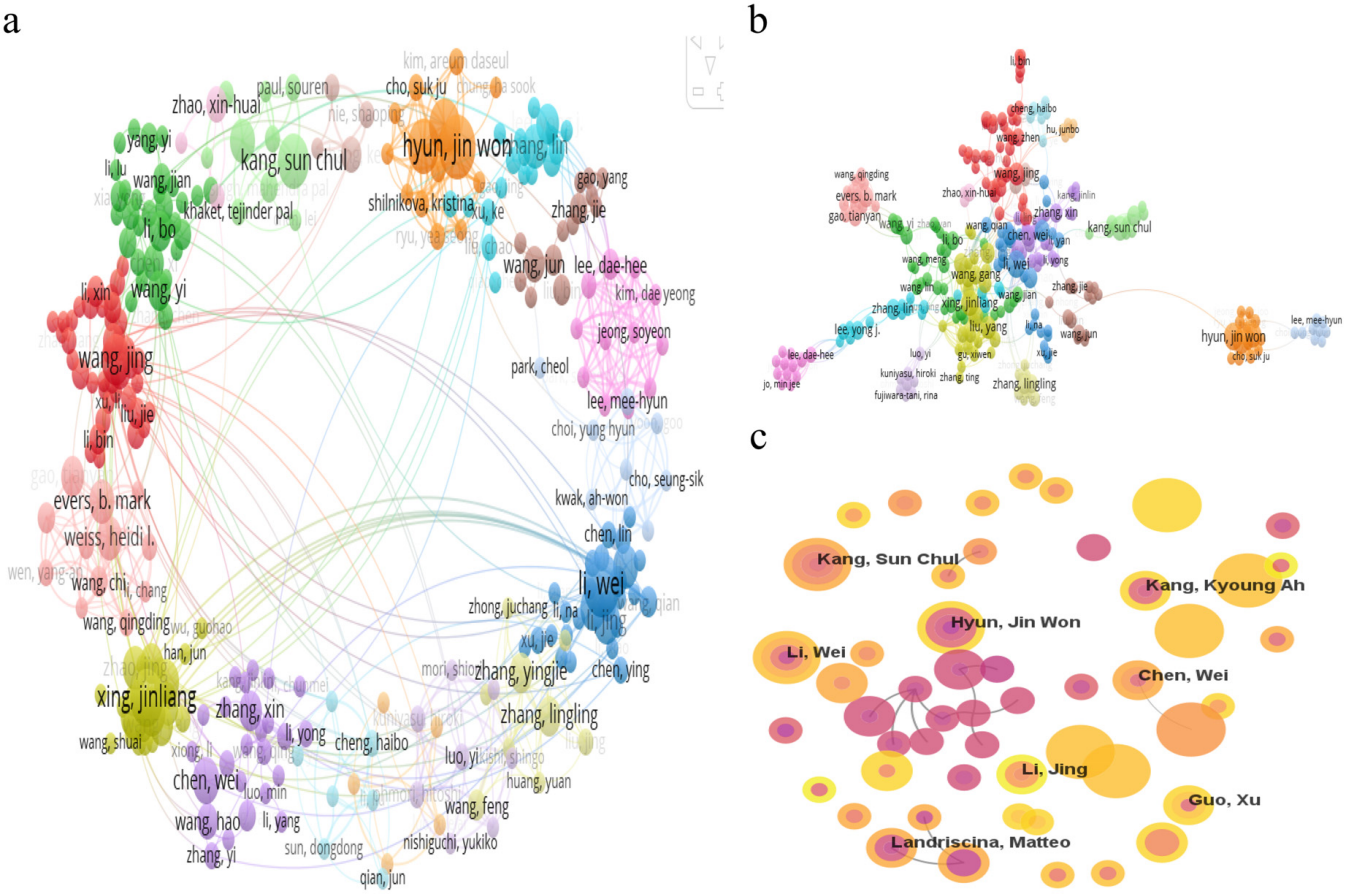
**(A)** Cooperation network visualization of authors in the field. **(B)** Cooperation network visualization of authors in the field based on LLR algorithm in the field. **(C)** Cooperation network of authors in the field.

Over the past decade, the field of mitochondrial function research in CRC has seen active involvement and significant contributions from 17,243 researchers. As detailed in **Figure 5A** and **Table 3**, Hyun Jin Won and Li Wei emerge as the most prolific researchers, each with 14 publications. To explore the collaborative relationships among these researchers, VOSviewer was used to generate the author network map shown in **Figures 5A, B**. This map clearly depicts the interconnectedness and interactions among authors engaged in CRC mitochondrial function research, shedding light on their academic collaborations and exchanges.

**Table 3:** Top 10 authors in terms of total publications and top 10 authors in terms of total citations.

Rank	author	documents	rank	co-cited authors	count
1	Hyun Jin Won	14	1	Ahmedin Jemal	393
2	Li Wei	14	2	Douglas Hanahan	273
3	Landriscina Matteo	12	3	Warburg O	179
4	Xing Jinliang	12	4	Wang Yan	163
5	Esposito Franca	11	5	LIU Y	145
6	Kang Kyoung Ah	11	6	WANG J	142
7	Kang Sun Chul	11	7	LI J	142
8	Piao Mei Jing	11	8	ZHANG L	141
9	Liu Yang	10	9	ZHANG Y	137
10	Szabo Csaba	10	10	Rebecca Siegel	125

A statistical analysis of co-cited authors was also conducted. Among the top 10 co-cited authors, Ahmedin Jemal leads with 393 co-citations, followed by Douglas Hanahan with 273 and Warburg O with 179 (as detailed in **Table 3**). To visually depict the citation relationships among these authors, CiteSpace was utilized for visual network analysis. **Figure 5C** illustrates the co-citation network map, where nodes represent authors, and the connections between nodes indicate the simultaneous citation of two authors in subsequent works. This map not only highlights the academic connections between these authors but also reveals the flow of knowledge and the academic legacy within the research field.

### 3.5 keyword clustering

**Figure 6:**
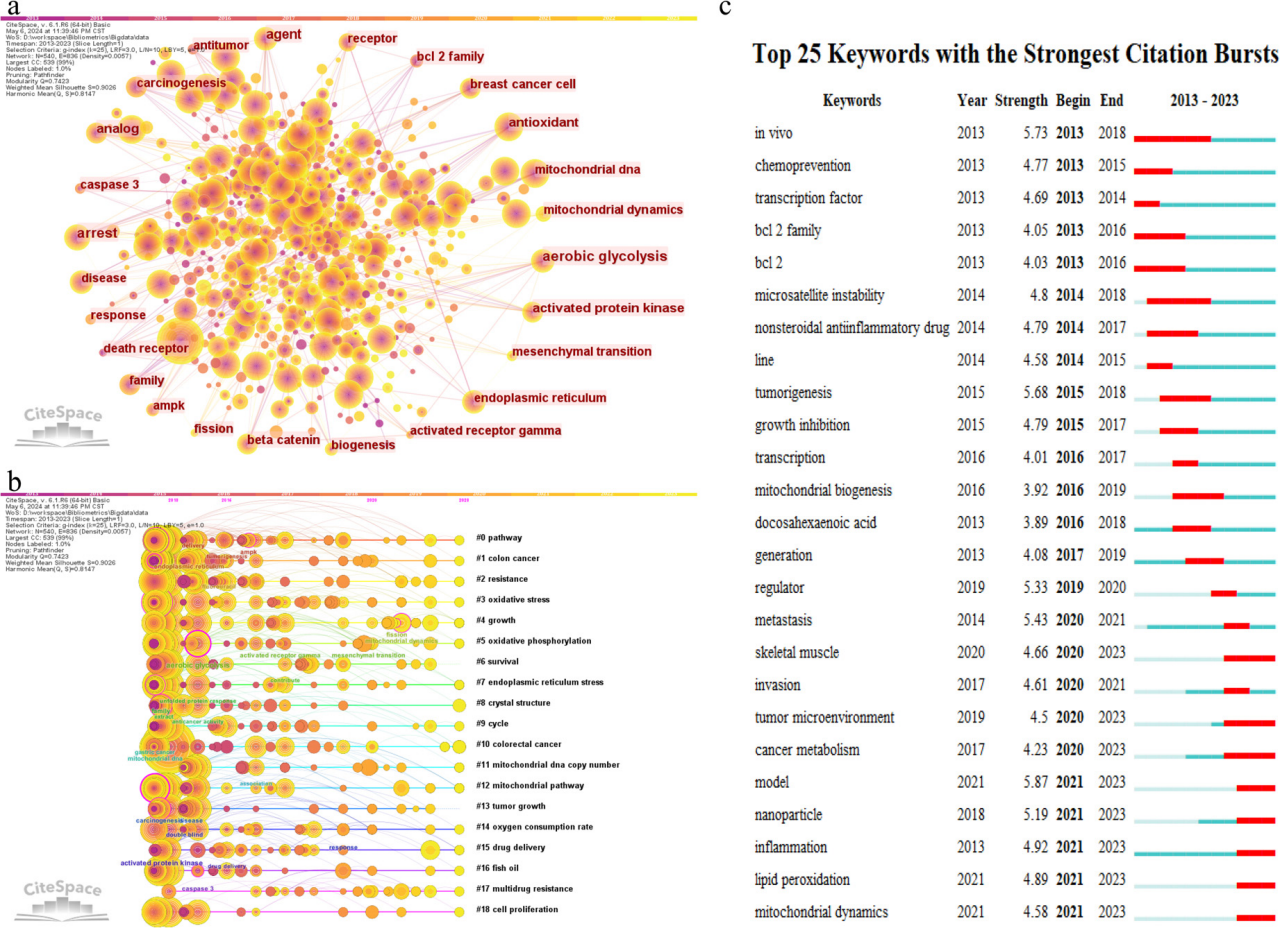
Co-occurrence network graph **(A)** and clustering timeline graph **(B)** of keyword generated by CiteSpace. **(C)** Top 25 most cited keywords.

**Table 4** lists the top 20 most frequently occurring keywords in CRC and mitochondrial research. “Apoptosis” emerges as the most prevalent keyword, appearing 1,112 times, followed by “colorectal cancer” (1,076 occurrences), “colon cancer” (608 occurrences), “expression” (474 occurrences), and “mitochondria” (451 occurrences). To visually illustrate the relationships and trends among these keywords, a visualization analysis was performed. As depicted in **Figure 6A**, setting the minimum occurrence of keywords to 5 allowed the successful clustering of 56 keywords into seven primary groups. These groups reflect various focal points within the research field and highlight the trending topics among researchers. **Figure 6A** presents a visualization map of keyword coverage, with colors mapped according to the average year of occurrence for each keyword, accompanied by a corresponding score. The map clearly shows that keywords like “colon cancer” and “oxidative stress” have gained prominence in mitochondrial-related research in recent years, indicating their status as current research hotspots and their potential to become future frontiers in the field. To further explore the intrinsic relationships and structures among these keywords, a keyword cluster visualization map was created, as shown in **Figure 6B**. This map groups the 56 keywords into 19 clusters, each centered around a core theme. These clusters include #0 pathway, #1 colon cancer, #2 resistance, #3 oxidative stress, #4 growth, #5 oxidative phosphorylation, #6 survival, #7 endoplasmic reticulum stress, #8 crystal structure, #9 cycle, #10 colorectal cancer, #11 mitochondrial DNA copy number, #12 mitochondrial pathway, #13 tumor growth, #14 oxygen consumption rate, #15 drug delivery, #16 fish oil, #17 multidrug resistance, and #18 cell proliferation. These clusters provide a comprehensive overview of the diverse research directions within CRC and mitochondrial studies, revealing potential connections and interactions among them. **Figure 6C** depicts the top 25 keywords with strongest citation bursts, with keywords such as TME, metabolism, nanoparticles, inflammation, lipid peroxidation, and mitochondria dynamics increasingly cited more in later years (2018–2023).

**Table 4:** Top 20 active keywords.

Rank	Keyword	Occurrences	Total link strength	Rank	Keyword	Occurrences	Total link strength
1	apoptosis	1112	2838	11	inhibition	257	813
2	colorectal cancer	1076	2282	12	proliferation	252	799
3	colon cancer	608	1401	13	growth	239	719
4	expression	474	1288	14	death	238	743
5	mitochondria	451	1248	15	cell	228	547
6	activation	358	1064	16	autophagy	226	678
7	oxidative stress	318	807	17	metabolism	224	569
8	cancer	303	647	18	mechanism	191	513
9	*in-vitro*	284	739	19	protein	165	455
10	pathway	262	825	20	p33	164	499

### 3.6 Journals and co-cited academic journals

Citation, defined as the frequency with which a piece of literature is referenced by other works, serves as a vital indicator of its influence and significance within the academic community. A higher citation count typically signifies a greater impact. In this study, two of the top 10 most cited references surpassed 1,000 citations. The most cited publication, “Proteogenomic Characterization of Human Colon and Rectal Cancer” by Zhang, B., Wang, J., et al., garnered 1,019 citations. Among these highly cited references, six were original research articles, while four were review articles. Geographically, four publications originated from the United States, and three from China, underscoring their substantial contributions to CRC proteomics research.

**Figure 7:**
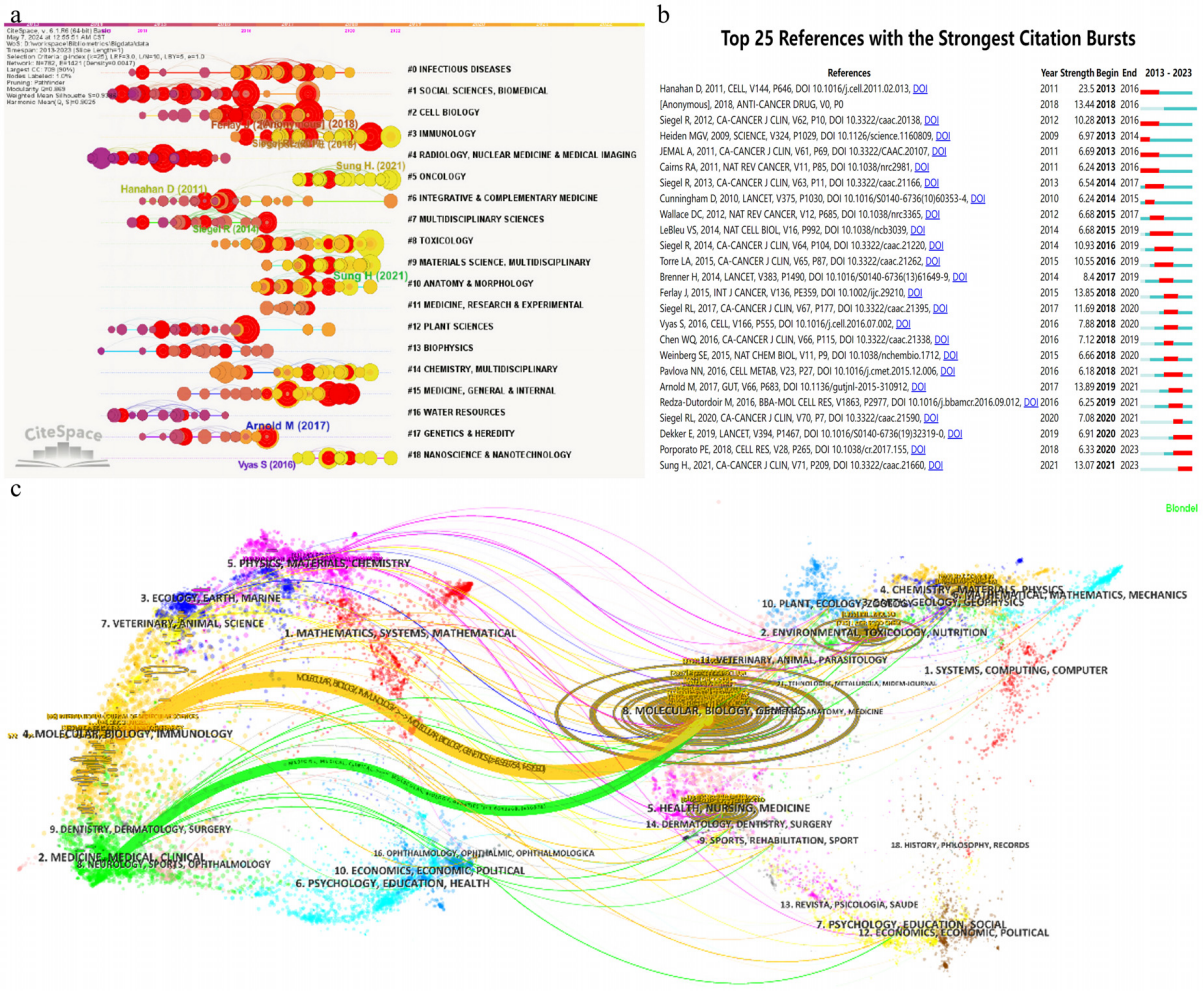
Images were all created using CiteSpace software: **(A)** Co-occurrence network clustering timeline graph. **(B)** Top 25 references with the strongest citation bursts. **(C)** Clustering relationship of journal analysis.

To further assess the academic impact of these references, CiteSpace software was utilized to perform burst detection and timeline view analysis, as shown in **Figure 7A**. The timeline view offers a clear visualization of academic progress and evolutionary trends in mitochondrial and CRC research, facilitating predictions of future research directions. In the graph, the time span is indicated at the top, with research clusters vertically arranged by size and the largest cluster positioned at the top. Larger circles represent references with higher citation counts or frequencies. The analysis identified 19 primary research clusters spanning multiple disciplines, including infectious diseases and nanoscience. Among these, Cluster #5, focusing on Oncology, is closely aligned with the subject of this study. In **Figure 7B**, burst detection highlights dynamic shifts in references for mitochondrial and CRC research, listing the 25 most cited references. The timeline is represented by a blue line, with red segments marking burst durations, indicating the start, end, and duration of each burst. The most cited reference, a 2017 publication by Arnold M., achieved a citation burst of 13.89. Additionally, three references continued to be cited through 2023, with two gaining significant attention starting in 2020.

Citation analysis deepens our understanding of the academic impact of human CRC proteomics research and its reach across various disciplinary fields. This analysis not only aids in evaluating the current research quality and level but also provides valuable insights for future research directions.

A detailed analysis of the co-citation map in **Figure 7C** reveals that the cited literature draws extensively from 7,606 distinct journals, with over 30 of these journals cited more than 660 times. The co-citation network is organized into three primary clusters, each marked by a distinct color, reflecting different research hotspots and trends. The top three most frequently cited journals are the “Journal of Biological Chemistry (JBC)” with 3,914 citations, “Cancer Research” with 3,869 citations, and “Cell” with 2,516 citations—all of which are prominent SCI top-tier journals, underscoring their significant influence and academic value in their respective fields. In top journals, as seen in **Table 5**, the International Journal of Molecular Sciences (IJMS) takes the lead with 97 published articles pertinent to the field, amassing 2024 citations. Close behind, PLoS One boasts 90 articles that have garnered 2632 citations. A point of particular interest lies in the fact that, while Cell Death & Disease and Oncotarget share the third position with 64 articles apiece, their respective articles have been cited 2834 and 2624 times, yielding an average citation rate per article that surpasses 40.

**Table 5:** Top 10 journals.

Rank	Journals	Publications	Times Cited	Average citation per article	H-Index
1	IJMS	97	2024	20.87	26
2	PLoS One	90	2632	29.24	31
3	Cell Death Dis	64	2834	44.28	28
4	Oncotarget	64	2624	41	30
5	Cancers	61	871	14.28	15
6	Oncol Rep	51	1306	25.61	23
7	Front. Oncol	50	1165	23.3	16
8	Front Pharmacol	48	1179	24.56	17
9	Molecules	48	1475	30.73	17
10	Sci. Rep	46	2255	49.02	24

The dual map overlay is an analyticalmethod that shows domain-level citation concentration with theirreference paths. **Figure 7C** depicts the citation linkages among journals, with citing journals positioned on the left side of the map and cited journals on the right. Each journal is annotated by its respective disciplinary affiliation, and the colored lines delineate the paths of citation. Within the dataset examined, two primary citation pathways are evident: the orange pathway signifies that journals focused on molecular/biology/immunology journals are regularly cited by journals in the field of molecular/biology/genetics journals; whereas the green pathway denotes that research published in medical/clinical journals is frequently referenced by studies in molecular/biology/genetics journals.

The co-citation map offers a comprehensive analysis of the academic journal network, revealing research hotspots and trends represented by different clusters. These insights enhance our understanding of current academic research priorities and provide valuable references and inspiration for researchers, thereby promoting the continued development of scientific inquiry.

## 4 Discussion

This study utilized bibliometric and information visualization methods to conduct a comprehensive and in-depth analysis of 2,823 papers on the association between mitochondria and CRC, published between 2013 and 2023. The analysis was structured around three key dimensions: quantitative, qualitative, and integrative, with the goal of creating a multi-dimensional view of the current research landscape in this field and offering insights into potential future research directions. The following sections will delve into the research findings, providing perspectives and insights from various angles.

### 4.1 General information

The research data clearly demonstrate the vitality and continuous growth of the field of mitochondrial and CRC research. The steady increase in annual publications reflects not only the rising interest and focus of researchers in this area but also underscores its significant value and vast potential for exploration in medicine and life sciences. Geographically, China leads the world in terms of publications, followed by the United States and India, highlighting the varying levels of research investment and output across different regions. However, despite China’s dominance in publication volume, its average citation rate lags behind that of the US and other countries. The US leads in average citations per publication, with the UK close behind, emphasizing the importance of high-quality research in enhancing academic influence and recognition. For China, the challenge lies in improving the quality and depth of research while maintaining high output levels. In terms of international collaboration, China and the United States have established the closest research partnership in this field, resulting in numerous significant research findings. The US has also engaged in extensive collaborations with other countries, further facilitating the global sharing and integration of research resources. At the institutional level, as seen in **Table 2**, Chinese research organizations demonstrate significant strength, with eight of the top ten institutions hailing from China, including Shanghai Jiao Tong University and China Medical University, both of which are at the forefront of this field due to their outstanding research achievements.

Regarding authorship, several prominent researchers have made substantial contributions to the development of the field through their academic expertise and dedication. Each of the top ten most active authors has contributed at least 10 high-quality papers, with Hyun Jin Won and Li Wei standing out as the most prolific, each with 14 publications. Co-citation analysis highlights Professor Ahmedin Jemal as a leading figure in the field, with his influential contributions and global impact on cancer research. His close collaboration with the American Cancer Society has not only deepened the understanding of global cancer trends but has also provided essential scientific evidence and support for developing cancer prevention and control strategies. In terms of journal performance, PLOS ONE has emerged as a key platform for disseminating research findings in this field, thanks to its extensive publishing resources and high-quality academic content.

### 4.2 Knowledge base

Co-citation analysis examines how frequently pairs of publications are cited together, offering insights into the interrelationships between literature and across fields (9). This approach not only reflects the academic influence of individual papers but also provides direct evidence of their scholarly value and importance. The higher the frequency of co-citation, the more significant the academic contribution. Researchers often focus on analyzing the top ten most frequently co-cited studies to quickly identify the core achievements and unresolved issues within a particular research field.

Among the top 10 co-cited studies, as seen in **Table 6**, groundbreaking discoveries have highlighted mitochondria as pivotal in the initiation and progression of CRC. In 2013, Csaba Szabo (10) led a study that elucidated the direct correlation between H2S production and mitochondrial function in colon cancer, revealing H2S’s multifaceted roles in promoting tumor growth and progression. The following year, Bing Zhang’s team (11) utilized TCGA data to unravel the proteomic complexities of CRC, with a focus on mitochondrial function-related genetic variations and protein expression changes, providing novel insights into the mechanisms underlying mitochondrial dysfunction in CRC. In the same year, Lu Yusheng (12) explored nitric oxide’s (NO) inhibitory effects on tumor metastasis, shedding light on the interplay between NO signaling pathways and mitochondrial function, offering fresh perspectives on CRC metastasis mechanisms. In 2015, Saad S. Dahham and colleagues (13) isolated β-caryophyllene from natural sources, demonstrating its ability to induce CRC cell apoptosis *via* a mitochondrial pathway, suggesting potential natural therapeutic approaches. Alain R. Thierry (14), in 2016, emphasized the significance of circulating DNA (cirDNA) in oncology, particularly the release of mitochondrial DNA during tumor cell apoptosis, introducing innovative concepts for CRC treatment strategies. Min Luo’s 2017 research (15) proposed a nanotechnology-based vaccine that leveraged the STING pathway to enhance anti-tumor immune responses, implicitly highlighting mitochondria’s vital role in immune cell activation and opening new avenues for CRC immunotherapy. In 2019, Xinming Jing and his team (16) elucidated the impact of hypoxic microenvironments on CRC biology, explaining how hypoxia modulates mitochondrial activity to influence various pathways, ultimately contributing to treatment resistance. Hai Hu and colleagues (17), although not directly focused on mitochondria, revealed the intricate interplay between endoplasmic reticulum stress and mitochondrial dysfunction in CRC progression. Dalia M. Kopustinskiene’s 2020 review (18) comprehensively explored how flavonoids modulate mitochondrial function to exert anticancer effects, also considering the role of gut microbiota, highlighting flavonoids’ potential in CRC prevention and treatment. Lastly, in 2022, Eric C. Cheung and his team (19) delved into the critical role of reactive oxygen species (ROS) in CRC, emphasizing mitochondrial-derived ROS as a driving force behind tumor growth, metastasis, and drug resistance, further underscoring the central importance of mitochondrial function in CRC pathophysiology.

**Table 6:** Top 10 cited document.

Rank	Title(Document Type)	First author	Times	Journal	Year	Country
1	Proteogenomic characterization of human colon and rectal cancer	Zhang Bing	1019	Nature	2014	the USA
2	Role of hypoxia in cancer therapy by regulating the tumor microenvironment	Jing Xinming	1004	Molecular Cancer	2019	China
3	The C/EBP Homologous Protein (CHOP) Transcription Factor Functions in Endoplasmic Reticulum Stress-Induced Apoptosis and Microbial Infection	Hai Hu	598	Frontiers in Immunology	2019	China
4	A STING-activating nanovaccine for cancer immunotherapy	Min Luo	586	Nature Nanotechnology	2017	the USA
5	Tumor-derived hydrogen sulfide, produced by cystathionine-β-synthase, stimulates bioenergetics, cell proliferation, and angiogenesis in colon cancer	Csaba Szabo	569	Biological Sciences	2013	the USA
6	Nitric Oxide Inhibits Hetero-adhesion of Cancer Cells to Endothelial Cells: Restraining Circulating Tumor Cells from Initiating Metastatic Cascade	Lu Yusheng	556	Scientific Reports	2014	China
7	Flavonoids as Anticancer Agents	Dalia M. Kopustinskiene	552	Nutrients	2020	Lithuania
8	Origins, structures, and functions of circulating DNA in oncology	Alain R thierry	515	Cancer and Metastasis Reviews	2016	France
9	The Anticancer, Antioxidant and Antimicrobial Properties of the Sesquiterpene β-Caryophyllene from the Essential Oil of Aquilaria crassna	Saad Sabbar Dahham	492	Molecules	2015	Malaysia
10	The role of ROS in tumour development and progression	Eric Chi Kin Cheung	439	Nature Reviews Cancer	2022	the UK

Collectively, these ten highly cited studies unravel the complex roles mitochondria play in CRC, from its initiation and progression to metastasis and treatment resistance. They highlight mitochondria’s intimate connections with ROS generation, endoplasmic reticulum stress, hypoxic microenvironments, metabolic reprogramming, and immune evasion. These insights not only deepen our understanding of CRC’s pathophysiological mechanisms but also pave the way for novel and effective therapeutic strategies, offering invaluable theoretical frameworks and potential targets.

### 4.3 Emerging topics

A thorough analysis of the literature and keywords related to mitochondria and CRC has highlighted oxidative stress, mitochondrial metabolism, and mitophagy as central areas of scientific focus. Current research is concentrated on uncovering fundamental mechanisms and developing precision-targeted applications for clinical cancer therapy as depicted in **Figure 6C**, encompassing aspects such as the tumor microenvironment (TME), cancer metabolic reprogramming, nanotechnology, inflammatory regulation, and mitochondrial dynamics.

The TME, a complex ecosystem consisting of tumor cells, immune cells, stromal cells, vascular networks, and various soluble factors and extracellular matrix components, involves intricate interactions like cell-to-cell communication, metabolic reprogramming, oxidative stress, and immune modulation (20). These interactions critically affect mitochondrial function in CRC cells, influencing tumor initiation and progression. The TME often operates under hypoxic conditions, compelling tumor cells to undergo metabolic reprogramming. In CRC, mitochondrial metabolic reprogramming stands as a crucial mechanism underlying tumor growth and development, Involves changes in the use of carbohydrates, lipids and amino acids (21). This metabolic shift not only satisfies the energy demands of rapid tumor cell proliferation but also supports the synthesis of essential biomolecules like nucleotides and lipids, thereby fueling tumor growth. The Warburg effect describes CRC cells’ preference for glycolysis even in the presence of sufficient oxygen, resulting in lactate production and rapid energy acquisition. Beyond increased glycolysis, abnormal mitochondrial metabolism is evident in the upregulation of glutamine metabolism, with CRC cells relying heavily on glutamine as a precursor for energy production and biosynthesis, thus promoting tumor growth and metastasis (22). The coordination of 1C metabolism between mitochondria and the cytoplasm also plays a role in CRC cell growth (23). Furthermore, studies have shown that silencing ammonia metabolism genes in CRC models increases intratumoral ammonia levels, directly inhibiting T-cell activity, exacerbating immune evasion, and indirectly impairing mitochondrial function by promoting oxidative stress (24). Research into mitochondrial metabolism in CRC continues to advance. Professor Chen Yingxuan’s team at Renji Hospital discovered that mitochondrial SIRT5 induces demalonylation of the GLUD1 lysine residue, significantly enhancing its enzymatic activity and increasing glutamine replenishment into the TCA cycle, providing crucial precursors for rapid CRC cell division and proliferation (25). This discovery establishes a theoretical and experimental basis for targeting SIRT5 as a potential therapeutic approach. Additionally, MTA1 has been shown to enhance colon cancer liver metastasis by driving mitochondrial metabolic reprogramming (26), and research indicates that age-related mitochondrial DNA mutations result in OXPHOS dysfunction, accelerating intestinal tumorigenesis and offering a novel perspective on the metabolic basis of tumorigenesis (27). Moreover, it has been shown that mitochondria from stromal cells are able to migrate into cancer cells with impaired mitochondrial function and restore their aerobic respiratory capacity. This cross-cellular transfer of mitochondria may represent a novel survival strategy for tumor cells in adverse environments, contributing to tumor proliferation.This finding provides insights into potential similar mechanisms in CRC, albeit requiring further investigation (28).

Current strategies for modulating mitochondrial metabolism focus on inhibiting key glycolytic enzymes, such as hexokinase and lactate dehydrogenase, or activating OXPHOS-related enzymes like pyruvate dehydrogenase to restore mitochondrial function and suppress cancer cell growth (29). Additionally, specific inhibitors targeting the glutamine metabolic pathway are under development (30). The use of Skp2 inhibitors to elevate IDH1 protein levels has been shown to redirect tumor cell metabolism toward a more functional TCA cycle, effectively curbing malignant proliferation (31). Ongoing development of targeted therapies for mitochondrial metabolic pathways has led to several drugs reaching clinical trials. For example, metformin, a mitochondrial ETC complex I inhibitor, has demonstrated anti-tumor effects in clinical trials for lung adenocarcinoma and ovarian cancer, showing promising application prospects (32). It provides important reference for the research and treatment of CRC.

Simultaneously, the hypoxic environment, nutrient deprivation, and accumulation of metabolic waste within the TME contribute to increased oxidative stress (33). Oxidative stress is an imbalance between oxidative and antioxidant effects in the body, favouring oxidation, leading to inflammatory infiltration of neutrophils, increased secretion of proteolytic enzymes, and the production of a large number of reactive oxygen radicals (ROS). Mitochondria, as the primary site of cellular energy production in eukaryotes, are both a major source and primary target of ROS (34). Oxidative stress impacts mitochondrial structure and function, modulating tumor cell survival, proliferation, differentiation, and apoptosis through the activation of signaling pathways and regulation of gene expression. It also inflicts oxidative damage on mitochondrial and intracellular DNA, proteins, and lipids. CRC cell mitochondrial DNA (mtDNA), lacking nuclear membrane protection, is particularly vulnerable to ROS, leading to mutations that compromise the integrity of the mitochondrial respiratory chain, reduce ATP production efficiency, and potentially facilitate tumor progression (35). Mitochondrial respiratory chain complexes I and III are significant ROS producers, and their dysfunction further elevates ROS levels by hindering electron transport (36, 37). In CRC, the expression and activity of antioxidant enzymes may be downregulated, impairing ROS clearance and exacerbating oxidative stress. ROS-induced DNA damage can activate proto-oncogenes and inactivate tumor suppressor genes, driving CRC initiation and progression. Furthermore, ROS modulates cell cycle regulation, apoptosis, and angiogenesis, influencing CRC invasion and metastasis. By stimulating tumor cells to release pro-angiogenic factors like VEGF, ROS promotes vascular endothelial cell proliferation, migration, and lumen formation, facilitating the construction of new vascular networks that supply oxygen, nutrients, and pathways for metastasis (38, 39). Oxidative stress levels are closely linked to CRC prognosis, with elevated oxidative stress potentially reducing chemosensitivity and radiosensitivity, thereby predicting poorer clinical outcomes. High expression of NOX4, an oxidative stress-related gene, is associated with a worse prognosis and shorter survival in patients with CRC (40). Elevated 8-OH-dG content in CRC tissue mtDNA suggests oxidative damage that may promote CRC development. Additionally, increased mtDNA copy numbers have been implicated in the progression of microsatellite-stable colorectal cancer by enhancing mitochondrial OXPHOS (41).

Current strategies for managing mitochondrial oxidative stress in CRC focus on antioxidation, anti-inflammation, and the mitigation of oxidative stress-induced cellular damage through the modulation of signaling pathways (42). Antioxidants such as CoQ10 and vitamins C and E play a pivotal role in neutralizing mitochondrial ROS, thereby protecting mitochondria from oxidative stress (43, 44). By inhibiting the production and release of inflammatory factors, these therapies can help alleviate inflammation-induced mitochondrial damage (45). Modulating mitochondrial-related genes and signaling pathways, particularly by activating the SIRT1/PGC-1α or NRF2 pathway, can enhance mitochondrial biogenesis, improve mitochondrial function, and reduce oxidative stress (5). Additionally, signaling molecules like LPA3 are crucial in regulating mitochondrial ADP-ATP exchange, with their activation playing a key role in maintaining mitochondrial homeostasis (46). Researchers are also developing novel anticancer drugs that target mitochondrial function, such as light-responsive nanoparticles, which effectively kill tumor cells by inducing mitochondrial oxidative stress and calcium overload (47). Antioxidants and mitochondrial-targeted drugs have shown promise in experimental models, with several advancing to clinical trials (48, 49). However, these therapies require further refinement to improve specificity and efficacy, as well as a thorough evaluation of their long-term safety and effectiveness (50). To fully understand the complex regulatory network of mitochondrial oxidative stress in CRC, researchers must integrate multi-omics data, including genomics, transcriptomics, proteomics, and metabolomics. In-depth investigations into the molecular mechanisms underlying mitochondrial oxidative stress will further elucidate the pathways of ROS generation and elimination and their effects on mitochondrial function and gene expression. These insights will be essential for designing and optimizing novel anticancer drugs, potentially leading to significant advancements in the prevention, diagnosis, and treatment of CRC by enhancing treatment efficacy and minimizing side effects.

In response to oxidative stress, CRC cells activate mitophagy as a protective mechanism to clear damaged mitochondria and mitigate cellular stress (51). Mitophagy, a key process for mitochondrial quality control, helps maintain cellular homeostasis by selectively targeting and degrading dysfunctional mitochondria. In CRC, abnormalities in mitophagy primarily involve both ubiquitin-dependent and ubiquitin-independent pathways (52). The ubiquitin-dependent pathway is mediated by PINK1 (PTEN-induced kinase 1) and Parkin (E3 ubiquitin ligase), which are key players in identifying and removing damaged mitochondria. A decrease in mitochondrial membrane potential leads to the accumulation of PINK1 on the outer mitochondrial membrane, where it activates Parkin. Parkin then ubiquitinates surface proteins on the mitochondria, recruiting autophagic receptors to initiate mitophagy (53). In CRC, disruptions in this pathway can result in either impaired or excessive mitophagy. In addition to the ubiquitin-dependent pathway, mitophagy can also be initiated through ubiquitin-independent mechanisms (54). These pathways involve direct interactions between specific receptor proteins, such as NIX, BNIP3, and FUNDC1, on the outer mitochondrial membrane and LC3, an autophagosome marker protein (54, 55). Abnormal expression or activity of these receptor proteins in CRC may disrupt normal mitophagy processes. Dysregulated mitophagy can contribute to tumorigenesis and cancer progression through various mechanisms (56). On one hand, tumor cells may enhance mitophagy to eliminate damaged mitochondria, thereby avoiding apoptosis and promoting survival and proliferation. On the other hand, excessive mitophagy may provide tumor cells with adaptability and resistance to treatment (57). CRC cells regulate mitochondrial quantity and quality through mitophagy, affecting cellular energy metabolism and contributing to the Warburg effect. Moreover, dysregulated mitophagy can interfere with normal cell cycle progression, inhibit apoptosis, and thus facilitate tumorigenesis (58). Recent research has identified key genes and signaling pathways that regulate mitophagy, highlighting new targets for the development of novel targeted therapies. Specifically, studies on mitophagy-related proteins, such as those involved in the PINK1/Parkin and BNIP3/NIX pathways, offer potential avenues to modulate mitophagy levels, thereby influencing tumor cell survival and proliferation, and providing new hope for cancer treatment (59, 60). Novel drugs that specifically regulate mitophagy are currently being developed. These include small molecules (e.g., Rapamycin, Chloroquine, Rotenone) and natural products (e.g., Curcumin, Resveratrol, Quercetin), which aim to influence the initiation and execution phases of mitophagy to inhibit tumor growth (4, 61, 62). Additionally, gene editing technologies, such as CRISPR/Cas9, can directly modify the expression of mitophagy-related genes, offering a more fundamental approach to regulating mitophagy for therapeutic purposes (63). The significance of mitophagy in CRC research is clear. It offers a new perspective on the molecular mechanisms of tumorigenesis and progression, as well as important targets for developing novel treatment strategies. However, the specific mechanisms of mitophagy in CRC are not yet fully understood, necessitating further research to unravel these processes, particularly the crosstalk and regulatory networks among different signaling pathways. By targeting key genes and pathways involved in mitophagy, it may be possible to develop highly effective, low-toxicity therapeutic drugs. Moreover, combining mitophagy modulation with chemotherapy, radiotherapy, immunotherapy, and other treatments could lead to improved therapeutic outcomes.

Multidrug resistance (MDR) presents a significant challenge in CRC treatment, with mitochondria playing a critical role in this phenomenon. The two primary mechanisms underlying MDR include the impairment of the mitochondrial apoptosis pathway and mutations in mitochondrial DNA (64). Impairment of the apoptosis pathway reduces tumor cells’ sensitivity to apoptotic signals, while mitochondrial DNA mutations disrupt normal mitochondrial functions, affecting cellular energy metabolism and apoptosis regulation. Current research focuses on mitochondrial DNA mutations, the regulation of hypoxia-inducible factor (HIF-1α), and alterations in key enzymes such as hexokinase-II (HK-II), and their effects on tumor cells’ response to chemotherapy. Studies have shown that high levels of HIF-1α and HK-II promote glycolysis in tumor cells under hypoxic conditions, thereby increasing drug resistance. Various drugs targeting mitochondrial function, including inhibitors of HIF-1α and HK-II, have demonstrated promise in reversing drug resistance *in vitro*. In addition, multiple signaling pathways and genes, such as PI3K/AKT/BAD and ERK/MAPK, along with drug resistance-related genes like mdr1/P-gp, have been identified as significant contributors to drug resistance mechanisms (65, 66). Notably, tetrandrine (Tet), a bisbenzylisoquinoline alkaloid derived from traditional Chinese medicine, has been found to inhibit P-gp function, thereby reversing MDR in CRC cells (67). Combining mitochondrial-targeted drugs with conventional chemotherapeutics has significantly improved the efficacy of CRC treatment (68). This approach not only enhances the sensitivity of chemotherapeutic agents but also reduces their side effects and mitigates the development of drug resistance. Nanoparticles, especially those capable of penetrating cell membranes and directly targeting mitochondria, offer innovative solutions for overcoming MDR (69). The complex interplay between CRC, mitochondrial function, and MDR highlights the need for further exploration to uncover novel therapeutic targets and inspire innovative strategies for CRC prevention and treatment.

In the field of immunology, mitochondrial dysfunction leading to excessive ROS production can directly impair immune cells and their functions (70). CRC cells under oxidative stress induce the expression of immune-suppressive molecules, such as programmed death-ligand 1 (PD-L1) (71). The interaction between PD-L1 and programmed death receptor 1 (PD-1) on T cells inhibits their activation and proliferation, thereby reducing their ability to eliminate tumor cells and facilitating immune evasion by the tumor. Mitochondrial dysfunction serves as a potential pathway for tumor immune evasion, as it can alter cell surface markers or release damage-associated molecular patterns (DAMPs) that may either trigger immune alert responses or be exploited by cancer cells to promote their growth and dissemination (72, 73). The decline in immune cells, coupled with the increase in immunosuppressive cells, collectively weakens the antitumor immune response, further exacerbating the vicious cycle of mitochondrial dysfunction. Moreover, research has shown that mitochondria from stromal cells can migrate into cancer cells with impaired mitochondrial function, restoring their aerobic respiratory capacity (57, 74). Although this mechanism requires further investigation in CRC (75), it offers insights into potential survival strategies used by tumor cells in adverse environments, contributing to tumor proliferation (28).

Mitochondria-targeted therapies in CRC hold immense potential, yet the path to clinical application is riddled with challenges. The complexity of biological mechanisms, variability in individual responses, and the difficulty in translating laboratory findings into viable treatments are significant obstacles. Utilizing advanced technologies such as nanotechnology and gene editing to create efficient, low-toxicity, and highly specific mitochondrial-targeted drugs offers the promise of revolutionizing CRC treatment. However, ensuring the safety of these therapies is critical, as they must precisely target tumor cells without causing irreversible harm to normal cellular functions.

## 5 Conclusions

In summary, this comprehensive review of the current research landscape on mitochondrial function in CRC highlights the necessity for further validation of the complex molecular mechanisms and signaling pathways involved. By employing advanced techniques in molecular biology, nanotechnology, and gene editing, it becomes possible to more accurately manipulate mitochondrial function and structure, paving the way for the development of more effective, safer, and targeted mitochondrial-based therapies. As ongoing clinical trials continue to integrate established molecular mechanisms with CRC drug targets, this research direction emerges as a promising frontier, poised to drive significant advancements in CRC treatment strategies.

## Data Availability

Publicly available datasets were analyzed in this study. This data can be found here: https://www.webofscience.com/ Web of Science Core Collection.
